# MMV-db: vaccinomics and RNA-based therapeutics database for infectious hemorrhagic fever-causing mammarenaviruses

**DOI:** 10.1093/database/baab063

**Published:** 2021-10-22

**Authors:** Taimoor Khan, Abbas Khan, Dong-Qing Wei

**Affiliations:** Department of Bioinformatics and Biological Statistics, School of Life Sciences and Biotechnology, Shanghai Jiao Tong University, 800 Dongchuan Road, Minhang District, Shanghai 200240, P.R. China; Department of Bioinformatics and Biological Statistics, School of Life Sciences and Biotechnology, Shanghai Jiao Tong University, 800 Dongchuan Road, Minhang District, Shanghai 200240, P.R. China; Department of Bioinformatics and Biological Statistics, School of Life Sciences and Biotechnology, Shanghai Jiao Tong University, 800 Dongchuan Road, Minhang District, Shanghai 200240, P.R. China; State Key Laboratory of Microbial Metabolism, Shanghai-Islamabad-Belgrade Joint Innovation Center on Antibacterial Resistances, Joint Laboratory of International Cooperation in Metabolic and Developmental Sciences, Ministry of Education and School of Life Sciences and Biotechnology, Shanghai Jiao Tong University, Shanghai 200030, P.R. China; Peng Cheng Laboratory, Vanke Cloud City Phase I Building 8, Xili Street, Nashan District, Shenzhen, Guangdong 518055, P.R China

## Abstract

The recent viral outbreaks and the current pandemic situation urges us to timely address any emerging viral infections by designing therapeutic strategies. Multi-omics and therapeutic data are of great interest to develop early remedial interventions. This work provides a therapeutic data platform (Mammarenavirus (MMV)-db) for pathogenic mammarenaviruses with potential catastrophic effects on human health around the world. The database integrates vaccinomics and RNA-based therapeutics data for seven human pathogenic MMVs associated with severe viral hemorrhagic fever and lethality in humans. Protein-specific cytotoxic T lymphocytes, B lymphocytes, helper T-cell and interferon-inducing epitopes were mapped using a cluster of immune-omics-based algorithms and tools for the seven human pathogenic viral species. Furthermore, the physiochemical and antigenic properties were also explored to guide protein-specific multi-epitope subunit vaccine for each species. Moreover, highly efficacious RNAs (small Interfering RNA (siRNA), microRNA and single guide RNA (sgRNA)) after extensive genome-based analysis with therapeutic relevance were explored. All the therapeutic RNAs were further classified and listed on the basis of predicted higher efficacy. The online platform (http://www.mmvdb.dqweilab-sjtu.com/index.php) contains easily accessible data sets and vaccine designs with potential utility in further computational and experimental work. Conclusively, the current study provides a baseline data platform to secure better future therapeutic interventions against the hemorrhagic fever causing mammarenaviruses.

Database URL: http://www.mmvdb.dqweilab-sjtu.com/index.php

## Introduction

The human-infecting seven mammarenaviruses associated with causing viral hemorrhagic fever are named *Lassa virus* (LASV), *Chapare virus* (CHAPV), *Lujo virus* (LUJV), *Guanarito virus* (GTOV), *Junín virus* (JUNV), *Machupo virus* (MACV) and *Sabiá virus* (SABV). Demographically, LASV and LUJV are considered indigenous to Africa, whereas CHAPV, GTOV, JUNV, MACV and SABV are common in American countries (1, 2). Mammarenavirus (mammalian arenaviruses) is an important genus of animal viruses and accommodates in the family called *Arenaviridae*. These are basically enveloped and spherical viral particles with a diameter of 50–300 nm (3, 4). The genome consists of two single-stranded (RNA) molecules known as L (large) and S (small) segments. Each genomic segment is responsible for the production of two different proteins, whereas the L segment serves as a genomic code for zinc-binding matrix protein (Z) and viral RNA-dependent RNA polymerase (L). Similarly, the S segment encodes an envelope glycoprotein precursor (GPC) and a nucleoprotein (NP) (5, 6). Additionally, two glycoprotein subunits called GP1 and GP2 of the spike are obtained after posttranslational cleavage during GPC synthesis (7, 8). The virions then utilize GP1 subunit to facilitate cell-surface receptor binding and enter the cell through endocytosis (9, 10). The other GP2 subunit mediates pH-dependent membrane fusion followed by uncoating and releasing viral ribonucleoprotein complexes inside the cell (11).

The mechanism of viral pathogenesis exhibits severe outcomes and a high fatality rate (12, 13) correlated with mammarenaviral hemorrhagic fevers. These viruses can be transmitted through aerosol or contact with infected person (3). The virus then gains systematic entry into the host lymphoid system with undetected pneumonic symptoms (14). The prominent targeting of macrophages (15, 16) and liver damage are considered a hallmark of pathogenicity (17, 18) during these human mammarenavirus infections. Similarly, compromised immune response function with secondary bacterial infections (19, 20) and leukocyte dysfunction in polymorphonuclear cells causing leukopenia (21, 22) are also associated with mammarenaviral infections. Other abnormalities, including the defective function of macrophages, depletion of the T- and B-lymphocytes, downregulated response of primary and secondary antibodies and high interferon (IFN) concentrations (23–25), has also been linked with hemorrhagic diseases.

The therapeutic approaches adapted for hemorrhagic fevers caused by different mammarenaviruses with related symptoms (26–28) vary with pathological conditions. Till now, there have been very few effective treatment options available to combat hemorrhagic fever in clinical setups. These treatment regimens include administering an adequate dosage of neutralizing antibodies during immune serum treatment (29) with related complications of transient cerebellar-cranial nerve syndrome (30, 31). Such passive antibody therapy options are also harbored by transfusion-borne diseases and require alternate treatments (32). The current anti-mammarenaviral therapy also includes the use of ribavirin (1-β-d-ribofuranosyl-1 H-1,2,4-triazole-3-carboxamide) with partial efficacy against some mammarenavirus infections. Meanwhile, the use of ribavirin is harbored by associated toxicity and adverse side effects, including severe anemia, thrombocytosis and birth-related defects in humans (33–35), Another candidate drug called T-705 (favipiravir) with targeted inhibition of target viral RNA synthesis and broad antiviral activity against RNA viruses (36, 37) is also used as a treatment option. Furthermore, no Food and Drug Regulatory Authority (FDA)-licensed vaccines are currently available to prevent mammarenavirus infections. The only designated live-attenuated vaccine that advanced to human clinical trials is called Candid 1 (Candidate no. 1), with efficacy against JUNV mediated infections (38, 39). Still, the continued search of potential vaccines expanded to several recombinant viruses, inactivated mammarenaviruses or alike particles (40) and other candidates tested in various animal models (41, 42) needs further evaluation with its potential therapeutic significance as a human vaccine.

For instance, the development of biological web databases for different diseases, i.e. breast cancer and cytomegaloviruses, are of great interest to researchers (43, 44). In this study, annotated data sets based on the genome and proteome analysis for seven species of human-infecting mammarenaviruses are presented. The analysis basically comprised of genome/proteome collection, immune-based epitopes prediction, vaccine designing and RNA-based therapeutics analysis. Additionally, the comprehensive therapeutic information is curated in the form of data sets available for free access to researchers. The extensive genomic and protein-specific investigation provides putative vaccine designs and RNA therapeutics options for utility in both advanced computational and experimental research. The novel platform, with protein-specific vaccine designs for each species and shortlisted potential siRNAs, microRNAs (miRNAs), and sgRNAs with all the necessary information, will aid in future therapeutic strategies against mammarenaviruses infections.

## Methodology

### Data collection

The whole-genome sequences (L and S segments) information used in this study were retrieved from the available online platform National Center for Biotechnology Information (https://www.ncbi.nlm.nih.gov/) (45), whereas the protein sequences (Z, L, G, N) of human-infecting mammarena species were collected from UniProt (https://www.uniprot.org/) (46). Further analysis was performed for the shortlisted human hemorrhagic fever-causing mammarena species. The accession and basic information of the genomic data set comprising of seven MMV species included in the study are given in Table 1. A list of protein sequences and accession IDs used in the study are also listed (Supplementary Sheet S1). The basic data information and sequences were then subjected to further analysis.

**Table 1.** Presenting genome-based accession information and other basic details of the cohort study MM species

Index	Virus name	Accession ID	Genomic length	Genome type
1	Lassa virus segment L	NC004297.1	7279 bps	linear RNA
	Lassa virus segment S	NC004296.1	3402 bps	
2	Chapare virus segment L	NC010563.1	7107 bps	linear RNA
	Chapare virus segment S	NC010562.1	3357 bps	
3	Lujo virus segment L	NC012777.1	7163 bps	linear RNA
	Lujo virus segment S	NC012776.1	3189 bps	
4	Guanarito virus segment L	NC005082.1	7081 bps	linear RNA
	Guanarito virus segment S	NC005077.1	3343 bps	
5	Junin virus segment L	NC005080.1	7114 bps	linear RNA
	Junin virus segment S	NC005081.1	3341 bps	
6	Machupo virus segment L	NC005079.1	7196 bps	linear RNA
	Machupo virus segment S	NC005078.1	3439 bps	
7	Sabia virus segment L	NC006313.1	7133 bps	linear RNA
	Sabia virus segment S	NC006317.1	3366 bps	

### Data processing

#### Epitopes prioritization

All the protein sequences of each mammarena species were initially scanned for immunogenic cytotoxic T lymphocyte (CTL) epitopes, B lymphocyte (B cell) epitopes, Helper T lymphocyte (HTL) epitopes and IFN-gamma-inducing peptides. The obtained epitope sequences for each species were further utilized to design highly immunogenic and antigenic epitopes-based *in silico* vaccines against each strain. To achieve the desired objectives, CTL epitopes for each protein of all species were predicted with the help of NetCTL 1.2 server (http://www.cbs.dtu.dk/services/NetCTL/) (47) and further characterized on the basis of combined score. The cut-off value used to predict CTL epitopes was set at 0.75. Similarly, B-cell epitopes prediction was carried out through ABCPred (http://crdd.osdd.net/raghava/abcpred/) server (48). The predicted linear B-cell epitopes were further filtered with a defined cut-off score of 0.5 in the process. Epitope ranking was done based on the binding score: the higher the score, the higher the probability of peptide inducing an immune response. Next, HTL epitopes (15mer) were obtained from the immune epitope database (IEDB) server (http://tools.iedb.org/mhcii/) (49) that showed good affinity for human Major Histocompatibility Complex (MHC) molecules (HLA-DRB1*01:02, HLA-DRB1*01:01, HLA-DRB1*01:04, HLA-DRB1*01:03 and HLA-DRB1*01:05), whereas the percentile ranking is inversely proportional to epitopes binding affinity and implies that a lower percentile rank would depict higher binding affinity (49).

Furthermore, IFN-γ-inducing peptides were filtered among these positive MHC-II peptides by employing IFNepitope web server (http://crdd.osdd.net/raghava/ifnepitope/) (50). The predictions were performed using IFNepitope server. Next, to select the best combination of epitopes that passes all experimental principles, antigenic epitopes were screened among the predicted cell epitopes by using Vaxijen v2.0 (51) with a default threshold of 0.4. To discriminate between allergens and nonallergens, AllerTOP v.2.0 (52) based on the k-nearest neighbors approach was used. The analyzed shortlisted peptides for each target protein with increased potential efficacy were included in further vaccine constructs.

#### Vaccine designing

Computational methods are of great interest to understand the molecular mechanisms of pathogenesis, drug resistance, and the development of novel therapeutics (44, 53–57). All the predicted epitopes for each protein were ranked accordingly based on the higher binding affinity. The final vaccine candidates were composed of adjuvant CTL, HTL (IFN +ive), and B-cell epitopes joined together by AAY, GPGPG, and KK linkers (58, 59), respectively. Herein, the vaccine sequences were further stabilized with added N-terminal human beta defensin-2 (hBD-2) sequence to ensure enhanced immunogenic response (60). The vaccine construct also needed to be antigenic for eliciting the proper immune response. For this purpose, the VexiJen server (http://www.ddg-pharmfac.net/vaxijen/VaxiJen/VaxiJen.html) (51) was employed to predict the vaccine’s antigenicity while keeping the threshold at the default 0.4. Another critical parameter, allergenicity, was predicted with the help of AlgPred server (http://crdd.osdd.net/raghava/algpred/) (61) at an accuracy of around 85%. Allergenic sequence can be identified when there is a score greater than the threshold (>−0.4). Physiochemical properties such as amino acid composition, molecular weight, theoretical pI, *in vivo* and *in vitro* half-life, instability index, aliphatic index, and grand average of hydropathicity (GRAVY) for experimental processing parameters were also employed to verify the vaccine. It was performed to unveil these properties for each vaccine construct by opting for an online webserver ProtParam (https://web.expasy.org/protparam/) (62). Furthermore, the 3D structures for all the vaccine constructs were predicted using the Robetta web server (https://robetta.bakerlab.org/) (63). In this procedure, the submitted sequences undergo domain-based initial recognition to forecast structure. This is followed by 3D modeling of submitted sequences depending on the type of templates available in the database. If matching templates are available, then comparative modeling is performed; otherwise, *de novo* modeling for 3D structures is performed. Finally, to address therapeutic implications, all the developed vaccine designs were listed and included as a separate data set for all the proteins of each MMV species.

#### Genome-based therapeutic RNA screening

The genome sequences (L and S Segments) for each species were further analyzed to predict siRNAs against each virus. Herein, virus-specific VIRsiRNApred server was employed with utilizing model 2 (64). The model is constructed based on integrated variable features, including hybrid nucleotide frequencies, thermodynamic properties, and binary pattern of already identified 1725 viral siRNAs. siRNAs that are highly efficacious with inhibition ≥50% were included. To evaluate immunomodulatory (IM) impact, the imRNA tool (65) was utilized to investigate IM and non-IM siRNAs. Similarly, putative miRNAs for Mammarena (MM) viruses were also predicted using two-step approaches. First, VMir algorithm (66) was used to predict precursor miRNA (pre-miRNA) hairpins by deploying default parameters. Second, the Mature Bayes tool (67) was used to identify mature miRNAs. All possible sgRNAs for MM viruses were also predicted using the ge-CRISPR tool (68) based on the Protospacer Adjacent Motif. This algorithm scans ‘NGG’ motifs in both forward and reverse strands of the genome and picks up putative sgRNAs located 20 nucleotides upstream of the motif. A regression-based algorithm was run on geCRISPR predictions to predict sgRNA with an efficiency of 0% to 100%.

### Development of database

The intricate process of database development was followed by using Apache HTTP (Hypertext Transfer Protocol) server v2.2.1.7 through open-source Linux, MySQL (My Structured Query Language) and PHP (Hypertext Preprocessor) to develop and deploy online the ‘MMV-db’ database. Front-end development and user interaction interface were designed using CSS (Cascading Style Sheets), HTML (Hypertext Markup Language), PHP, and JavaScript, which also provides searching and downloading function. For back-end development of the database WAMP (Windows, Apache, MySQL, PHP) server accompanied by scripting in environments like HTML and PHP was used. Data storage, manipulation and retrieving from the databases were managed through MySQL to confer complete control over the web contents.

## Results and discussion

**Figure 1.**
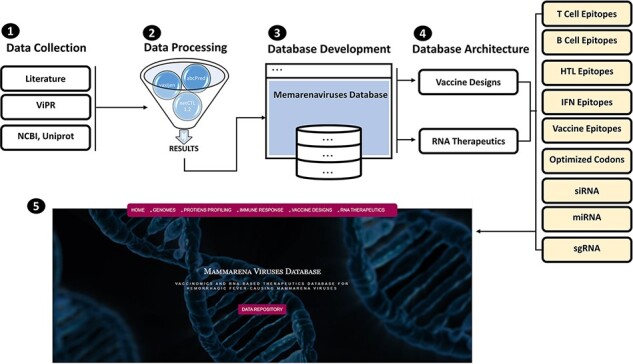
(From 1 to 5) represents the general schematic workflow and different steps followed in the development of mammarenavirus database.

MMV-db focus spanned from basic protein features profiling to advanced epitopes-based vaccine designs and RNA-based therapeutics for all human-infecting MM viruses. This database is a collective platform for a total of seven hemorrhagic fever-causing-related mammarenaviruses. The database includes multiple-features profiling, including genome and proteins sequences, vaccine designs and therapeutic RNAs information represented in different tabs of the developed online platform. The overall workflow of the strategy, including the data source utilized in the design of this database, has been given in Figure 1.

### Immune-based features profiling for mammarenaviruses

**Figure 2.**
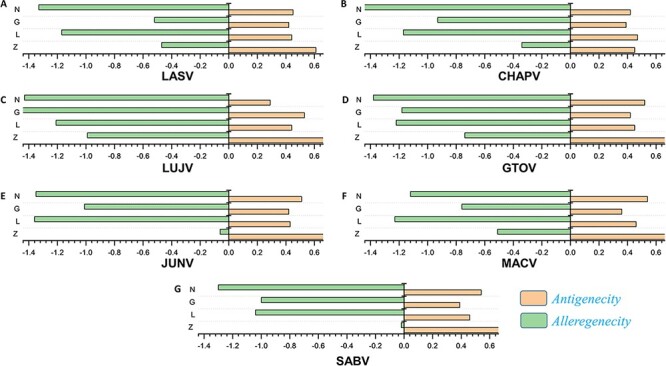
(A–G) Represents the antigenicity and allergenicity scores for whole-protein sequences in the individually studied mammerenaviruses.

The antigenic and nonantigenic proteins for each species were identified with a VaxiJen threshold scoring system (51). The server utilizes an alignment-free, covariance-based approach with a focus on the properties of amino acids (51). We choose the target organism as a virus and initiated the analysis with a sequence-based output with default criteria. The antigenicity profiling of all the proteins was performed including all the mammarena viruses. The cut-off value of 0.4 was used as an indicative threshold to differentiate between potential viral antigenic and nonantigenic proteins (51). Proteins were further subjected to allergenicity prediction analysis. The performed allergenicity check helps to prevent any possible allergic responses in the host (69). The server algpred v. 2.0 (70) was utilized to predict the allergenicity of the proteins, whereas a score greater than the threshold (>−0.4) represents allergenic sequences [49]. The input sequence was added as a single letter amino acid code, while the selected prediction approach was an amino acid composition-based Support Vector Machines (SVM) module (70). The immune-based analysis of antigenicity and allergenicity was performed to profile each species-specific protein. The output data were arranged on the basis of obtained scores to differentiate between antigenic, nonantigenic, allergenic and nonallergenic proteins. The antigenicity and allergenecity profiles for each of the four (Z, L, G and N) specific proteins of all species are shown in Figure 2.

**Figure 3.**
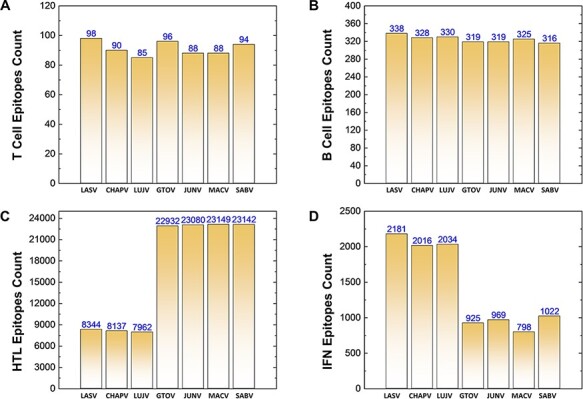
(A–D) Demonstrates the total identified number of predicted immune epitopes for each specific protein; (A) represents the total number of T-cell epitopes, (B) represents the total number of B-cell epitopes, (C) represents the total number of HTL epitopes, while (D) represents the total number of IFN epitopes predicted for each protein for the seven mammarena species, respectively.

Similarly, a total of 639 CTL epitopes, 2275 B-cell epitopes, 116 746 HTL epitopes and 9945 IFN epitopes were analyzed for all the mammerenavirus species. The predicted epitopes were further classified on the basis of species-specific proteomes. The total count of whole proteome-specific T-cell, B-cell, HTL and IFN-inducing epitopes were calculated for each studied species and presented as shown in Figure 3A–D, respectively.

### Immunogenic and putative vaccine epitopes prediction

**Figure 4.**
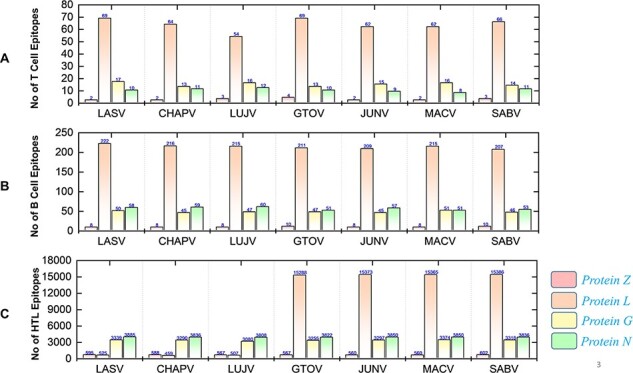
Demonstrates the total identified number of classified immune epitopes for each specific protein; (A) represents the number of specified T-cell epitopes, (B) represents the number of B-cell epitopes and (C) represents the number of HTL epitopes predicted for each protein for the seven mammarena species, respectively.

The immunogenic and potential vaccine epitopes screening was performed in a sequential manner for all the seven mammarena viruses i.e. LASV, CHAPV, LUJV, GTOV, JUNV, MACV, and SABV. Moreover, the order of proteins in the results is presented as Z, L, G (representing GPC) and N (representing NP) for each species. The prediction of T-cell, B-cell and HTL epitopes obtained after protein sequence-based analysis are presented with the total number of each type of epitope for a specific protein in individual species (Figure 4).

**Figure 5.**
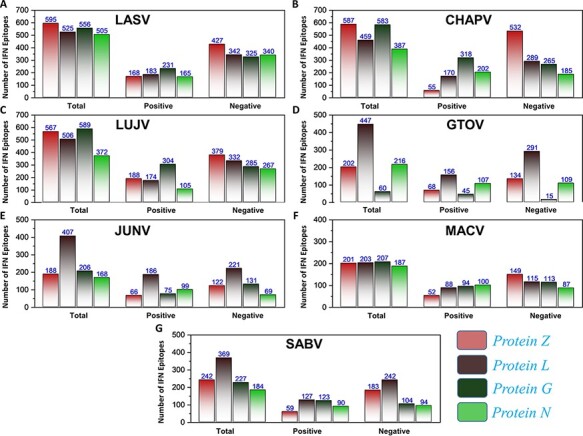
(A–G) Represents the total number of predicted IFN epitopes along with positive and negative IFNs for each protein of the seven MMVs, respectively.

First, the prediction of potential CTL epitopes related to the four L, S, G and N was performed. For this purpose, Net CTLpan *v*1.2 was utilized (47), and predictions were performed using 12 different supertypes of human leucocyte antigen with the rest of the default parameters. The sequences of the peptides having a % Rank <1% (*<*E) were chosen as MHC binders according to the given selection criteria of the webserver. The protein-specific T-cell epitopes (Supplementary Sheet S2) were further classified for each species, as shown in Figure 4A. Moreover, linear B-cell epitopes also work as antigen and interact with B-cell receptors. Identification of B-cell epitopes is vital to generate a protective host antibody response. Correspondingly, total B-cell epitopes were predicted (Supplementary Sheet S3) by using ABCpred server (48) and further classified for each protein as shown in Figure 4B. Likewise, HTL epitopes based on protein sequences were also predicted using the IEDB MHC-II binding prediction tool (49). Using the selection criterion mentioned in methodology, MHC-II binders were predicted, and a collection of these epitopes were further screened for potential IFN induction property. The total number of HTL epitopes for each protein of the examined species is shown in Figure 4C. Furthermore, the IFN-γ produced by T-helper cells is vital in clearing the virus; therefore, selected HTL epitopes were subjected to shortlisting based on IFN-γ induction. The selected HTL epitopes, as shown in Supplementary Sheet S4, were further characterized for IFN-inducing positive and negative epitopes for all proteins of each species as shown in Figure 5.

Further evaluation made by subjecting epitope sequences to antigenicity and allergenicity prediction helped to identify putative vaccine candidates. After the extensive analysis, putative vaccine epitopes from all groups were shortlisted, with qualifying parameters for each protein of all species included in further vaccine designs.

### Construction of multi-epitope vaccines

**Figure 6.**
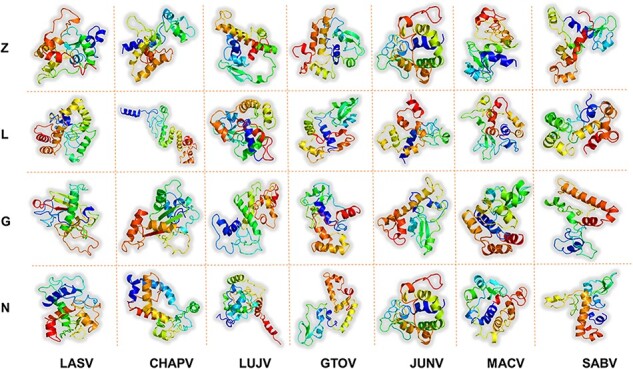
Represents the refined 3D structures of MEV designs for each protein of all the studied mammarenavirus species. x-axis includes names of the MMV species, while y-axis shows the vaccine-targeted proteins.

Vaccines therapy based on targeting large protein or whole organisms is considered highly effective in reducing the burden of viral infections and associated mortality (71) around the world. However, it is also harbored by a high antigenic burden of such vaccines, imprecise immunological responses and associated reactogenic reactions (72, 73). Alternatively, peptides-based vaccine designs are more appropriate options for clinical use. The feasibility of peptide vaccines is reflected by reduced production time and cost, robust immune-specific response provoking, lowered risk of antigen-induced allergies and flexibility to varied antigens (74, 75). However, the administration of peptide vaccines needs appropriate adjuvants to ensure immunogenic nature (76). This also shelters the inclusion of feebly antigenic individual peptides and enhances immunogenicity in the overall multi-epitope-based vaccine designs (77). Herein, the designed Multi-epitopes Vaccine Construct ( MEVC) sequences based on shortlisted T-cell, HTL and B-cell epitopes with fulfilled selection parameters for robust immune response are shown (Supplementary Sheet S5). The topographical arrangement of the epitope-based vaccines was further equipped with appropriate linkers. Different types of linkers such as EAAK, GPGPG, AAY and KK were used to join these small peptides together and design a full-length MEVC. The linkers utilized in MEVC constructs are related to the facilitation of epitopes display and efficient induction of immune response (78). Altogether, the linkers hindered epitopes folding (58), and enhanced adjuvant immunogenicity (59) was considered during MEVC designing. Thus, N-terminal of the modeled peptide vaccines was furnished with a nontoxic adjuvant called human beta defensin-2 (hBD-2) with added EAAAK linker. The adjuvant (hBD-2) has a regulated expression and also potentiates immune response against attached antigen [83]. The amino acid sequences for each MEVC were further verified for antigenic and allergenic properties (Supplementary Sheet S6) to elude any adverse immune reaction in further experimental designs. The systematic analysis was followed for all the potential vaccine construct designs for each protein of the seven mammarena species. The final MEVC designs were also subjected to codon optimization using the JCAT server (79), and explored DNA sequences are presented (Supplementary Sheet S6) for further utility in procedures of *insilco* and experimental cloning. Furthermore, the robetta-predicted 3D structures of final vaccine constructs for each protein of the seven mammarena species are shown in Figure 6.

### Analyzing physicochemical properties

The ProtParam server (62) was utilized to determine the MW and Isoelectric Point (pI) of the MEVC sequences designed against each protein, respectively. The pI values suggested a more suitable acidic nature of the candidate vaccines. Similarly, the distribution number of positively and negatively charged residues, extinction coefficient of the protein in water, *in vitro* half-life of the vaccine constructs in mammalian reticulocytes and *in vivo* in yeast and *Escherichiacoli* were also listed. Furthermore, the aliphatic index and GRAVY value of the proposed vaccine were determined, which reflects the stability of the protein with temperature change. Similarly, the obtained negative GRAVY value indicates hydrophilic nature and improved interactions of the vaccines with nearby water molecules. The physiochemical properties of each vaccine with all the details are provided in Supplementary Sheet S6.

### Genome-based prioritization of candidate therapeutic RNAs

The potential therapeutic RNAs were also identified for all the seven species of human-infecting mammarenaviruses. The genome-based analysis was performed for each species as L, S segments and whole genome. The predicted therapeutic RNAs were further characterized on the basis of efficacy scores, including siRNAs, IM/non-IM siRNAs, miRNAs and sgRNAs. The data are also presented in different tabs with the accessible necessary information in the online resource.

#### SiRNAs

**Figure 7.**
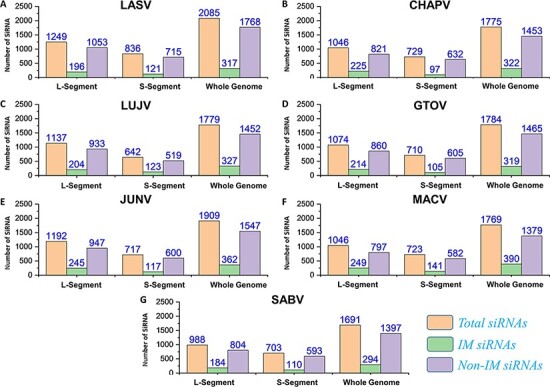
(A–G) Represents number of total SiRNA’s and classified IM/non-IM siRNAs identified for genomic segments L, S and whole genome of each mammarenavirus.

Silencing viral genes by RNA interference is an excellent alternative therapeutic option (80). Regarding this, all possible efficient siRNAs targeting genes of the mammarena species were investigated. The analysis performed was specified to L, S and whole-genome segments for all the studied mammarenavirus Species. The analysis involved a collective number of 2085, 1775, 1779, 1784, 1909, 1769 and 1691 siRNAs for each of the species, respectively. This was achieved after deploying the VIRsiRNA algorithm (64) with identifying potent siRNAs of more than 50% predicted ability of inhibition. Following, all these siRNAs with higher efficacy scores were further subjected to IM potential analysis by using the iMRNA tool (65). Total siRNAs, immuno-modulatory (IM) SiRNAs and non-IM SiRNAs with predicted counts for L, S and whole-genomic segments specific to each species are presented in Figure 7A–G. All the relevant information of the total siRNAs and their therapeutics-related IM potential scores (Supplementary Sheet S7) are also available in separate tabs of the MM-db virtual resource.

#### MicroRNAs

**Figure 8.**
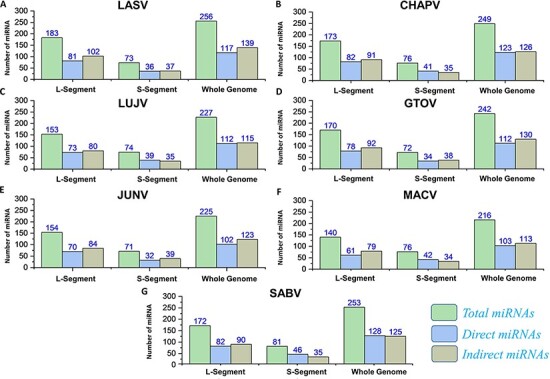
(A–G) Represents the number of total miRNAs and orientation-based classified direct/indirect mature RNAs identified for L, S and whole-genome segments of each mammarenavirus.

The applications of miRNA interference-based therapies spans from targeting a single gene to complex cellular pathways (80). In our data, all the pre-miRNAs along with mature miRNAs (3p and 5p) were also identified by deploying VMIR tool (Analyzer and Viewer) (66). Complete information of the hairpin sequence, its length, start-end location, mature miRNA sequence, orientation and scores were retrieved. This analysis revealed a total number of 256, 249, 227, 242, 225, 216 and 253 potential miRNAs for whole genome of each mammarenavirus. The mature miRNAs (3p and 5p) (Supplementary Sheet S8) obtained after mature Bayes tool analysis (67) were further classified on the basis of orientation as direct or reverse. The computed number of orientation (direct and reverse) specific and total miRNAs for each L, S and whole-genome segments of mammarena species are shown in Figure 8. The resulting mature miRNAs with mentioned orientation are also available in the data sets provided on the server.

### Putative sgRNAs

**Figure 9.**
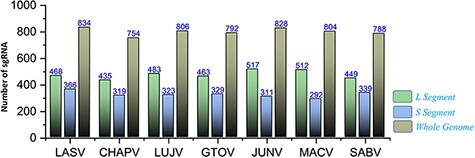
Represents the total number of sgRNAs identified for L, S and whole-genome segments respective to each of the seven mammarenavirus.

The recently advanced biological strategies also involve sgRNA-based therapeutic interventions (81). Significantly, putative efficient sgRNAs were also enlisted after utilizing the ge-CRISPR-based analysis (68). The useful information regarding sgRNA sequences in 5ʹ to 3ʹ direction, start and end positions, Guanine-Cytosine (GC)%, Protospacer Adjacent Motifs (PAM) and predicted sgRNA efficiency is also presented in Supplementary Sheet S9. The number of predicted sgRNAs for the different genomic segments of each species is shown in Figure 9. The data are also made easily available for researchers in the online platform to be utilized in the identification of CRISPR targets, including efficient sgRNAs in further experimentation designs against mammarenaviruses.

## Conclusion

Despite the advancement in the field of biology, many viral disease still suffer from the lack of proper treatment i.e. vaccines or drugs. Our investigations present 28 different multi-epitopes-based protein-specific vaccine designs with a critical role in immune response induction against seven different mammarena species. Further exploration of physicochemical properties also suggests experimental feasibility of the vaccines. However, the efficacy and safety of the highly specific MEVC candidates need further demonstration through lab experiments and remain elusive. The developed online platform also offers RNA-based therapeutic options for further investigations against human-infecting MMVs. Altogether, MMV-db offers a novel predisposed source of advanced multi-epitopes-based vaccines and RNA-based therapeutics against MMV species pathogenic to humans.

## Supplementary Material

baab063_Supp

## Data Availability

All the data are given in the Supplementary materials.
